# Reframing the epidemiological transition as increasing returns to tackling aging-related diseases

**DOI:** 10.1038/s43587-026-01210-2

**Published:** 2026-09-02

**Authors:** Julian Ashwin, David E. Bloom, Naomi Lee, Peter Piot, Andrew J. Scott

**Affiliations:** 1 Department of Economics, Maastricht University, Maastricht, the Netherlands; 2 Department of Global Health and Population, Harvard T.H.Chan School of Health, Harvard University, Cambridge, MA USA; 3 National Bureau of Economic Research, Cambridge, MA USA; 4 Centre for Economic Policy Research, Paris, France; 5 IZA, Luxembourg, Luxembourg; 6 Ellison Institute of Technology, Oxford, UK; 7 Department of Infectious Disease Epidemiology and Dynamics, London School of Hygiene and Tropical Medicine, London, UK; 8 Department of Economics, London Business School, London, UK

**Keywords:** Diseases, Policy

## Abstract

Global life expectancy has risen since 1973 from 58 to over 73 years, while the share of people aged 65 years and older has doubled to 10% and is projected to reach 21% by 2073. This shift is linked to an epidemiological transition from infectious to chronic disease. Here, using Global Burden of Disease data, we reframe this transition by statistically grouping diseases on the basis of their impact over the life cycle, yielding four clusters: infant, early-adult, later-adult and aging-related. We show aging-related diseases are the largest part of the current global disease burden and the greatest lifetime burden for a newborn, even in low-income countries. Aging-related diseases possess two distinctive properties: a tight link between mortality and morbidity, and increasing returns, whereby reductions in their prevalence makes further gains even more attractive. The implications of this recasting of the epidemiological transition for health systems and aging research are discussed.

## Abstract (web summary)

Reframing diseases by when they occur over the lifespan reveals that aging-related diseases now dominate the global burden in every income group. They also uniquely show increasing returns, meaning that the more we tackle them, the greater the gains.

## Main

Between 1973 and 2023, global life expectancy at birth increased from 58 to more than 73 years^1^, an average improvement of 3 years every decade (Table 1). While the rate of increase varied across countries, life expectancy rose everywhere. In 1973, Andorra had the highest life expectancy (75 years), and in 2023, Monaco did, at just over 86 years. In 1973, Mali had the lowest life expectancy (35.1 years), while today Nigeria does, at 54.5 years.

**Table 1:** Global life expectancy, survival rates and healthy life expectancy: 1973–2023

Income group	Year	Life expectancy at birth (years)		Survival rate			Year for final column	Proportion of life that is healthy
			0–65	0–70	0–80	65–80		
Global	1973	57.7	0.553	0.460	0.224	0.405	1990	0.862
	2023	73.2	0.772	0.699	0.473	0.613	2023	0.853
High income	1973	70.7	0.749	0.652	0.362	0.484	1990	0.862
	2023	80.2	0.864	0.811	0.649	0.751	2023	0.846
Upper-middle income	1973	59.1	0.577	0.470	0.217	0.376	1990	0.871
	2023	75.2	0.833	0.764	0.518	0.622	2023	0.861
Lower-middle income	1973	50.4	0.445	0.357	0.151	0.339	1990	0.860
	2023	68.3	0.735	0.640	0.371	0.504	2023	0.858
Lower income	1973	44.8	0.378	0.302	0.131	0.346	1990	0.861
	2023	64.5	0.669	0.571	0.319	0.476	2023	0.859

Income group is defined by 2023 World Bank status. Survival rate is the probability of an individual surviving from one age to the other. Life expectancy and survival rates are from the United Nations World Population Prospects. The final column is the ratio of healthy life expectancy to life expectancy calculated from the GBD study (Methods).

If old age is defined as 65 years and older (a somewhat arbitrary threshold^2^), three-quarters of babies born in 2023 are expected to become old, ranging from nine out of ten in high-income countries to two out of three in lower-income countries. Furthermore, young people are now likely to live substantially beyond 65 years; nearly two out of three born in high-income countries and nearly one in three in lower-income countries can expect to live to 80 years (assuming no further mortality improvements). In 1973, no country had a life expectancy above 80 years, whereas by 2023, 42 countries did and the United Nations predicts 149 such countries by 2071^1^.

Combined with falling fertility, these trends are producing substantial shifts in the global age structure. In 1970, people aged 65 years and over accounted for around 5% of the world population, today that statistic has doubled to reach 10%, and by 2073 is projected to be 21%.

Associated with these trends is the concept of an epidemiological transition^3^, which is defined by a shift in the disease burden away from infectious toward degenerative/chronic diseases, with the latter having risen from 42% of the global disease burden in 1990 to 64% in 2023. Related to this shift in the disease burden is the fact, shown in Table 1, that the ratio of healthy life expectancy to life expectancy declined slightly across all income groups over this period, implying that life expectancy gains are resulting in more years spent in poor health and thus expanding morbidity.

In this Analysis, we reframe the epidemiological transition using Global Burden of Disease (GBD) study data to statistically define diseases by when they have their greatest impact over the life cycle. In doing so, we arrive at four distinct disease clusters: infant, early adult, late adult and aging-related. By defining the disease burden relative to the life cycle, we more closely integrate the epidemiological transition with the demographic transition, which is defined by changes in the age structure of the population. Our statistical approach enables us to quantify current and future mortality and morbidity trends across these clusters, but more crucially, identifies two unique properties of aging-related diseases (which we refer to as ‘complementarity’ and ‘increasing returns’) with important implications for the future of policy, health systems and medical research. As a result, our reframing of the epidemiological transition stresses that the issue of whether the etiology of a disease is chronic, noncommunicable or infectious is secondary to whether it is aging-related.

## Results

### Data and classification

Three motivations drive our life-course approach to allocating diseases into distinct clusters. First, as the median population age increases, the disease burden changes because the incidence of individual diseases varies with age. Second, life expectancy gains may themselves occur through unequal reductions in the disease burden. For instance, between 1990 and 2023, the share of the global burden of disease (based on disability-adjusted life years or DALYs) declined from 3.5% to 0.45% for measles, from 0.9% to 0.36% for whooping cough (pertussis) and from 0.86% to 0.04% for tetanus. These changes will increase life expectancy and change the relative importance of infant diseases. Finally, motivated by geroscience^4–6^, we are interested in the possibility that at older ages the aging process itself becomes central to health.

#### Definition of disease clusters

To link diseases to stages of the life cycle, we pursue a statistical rather than biological approach. Specifically, we use a *K*-means++ unsupervised clustering algorithm^7^ to allocate diseases to distinct groups on the basis of their peak prevalence, as measured by the number of DALYs lost per person at each age. Our source data for diseases is the GBD study^8^, covering 304 diseases and injuries across 204 countries between 1990 and 2023. To construct population data by age group we used the 2024 United Nations’ World Population Prospects^1^. The Methods section describes our data and definitions. We standardize DALY rates to have zero mean and unit standard deviation so that diseases are clustered solely on their age profile.

**Fig. 1:**
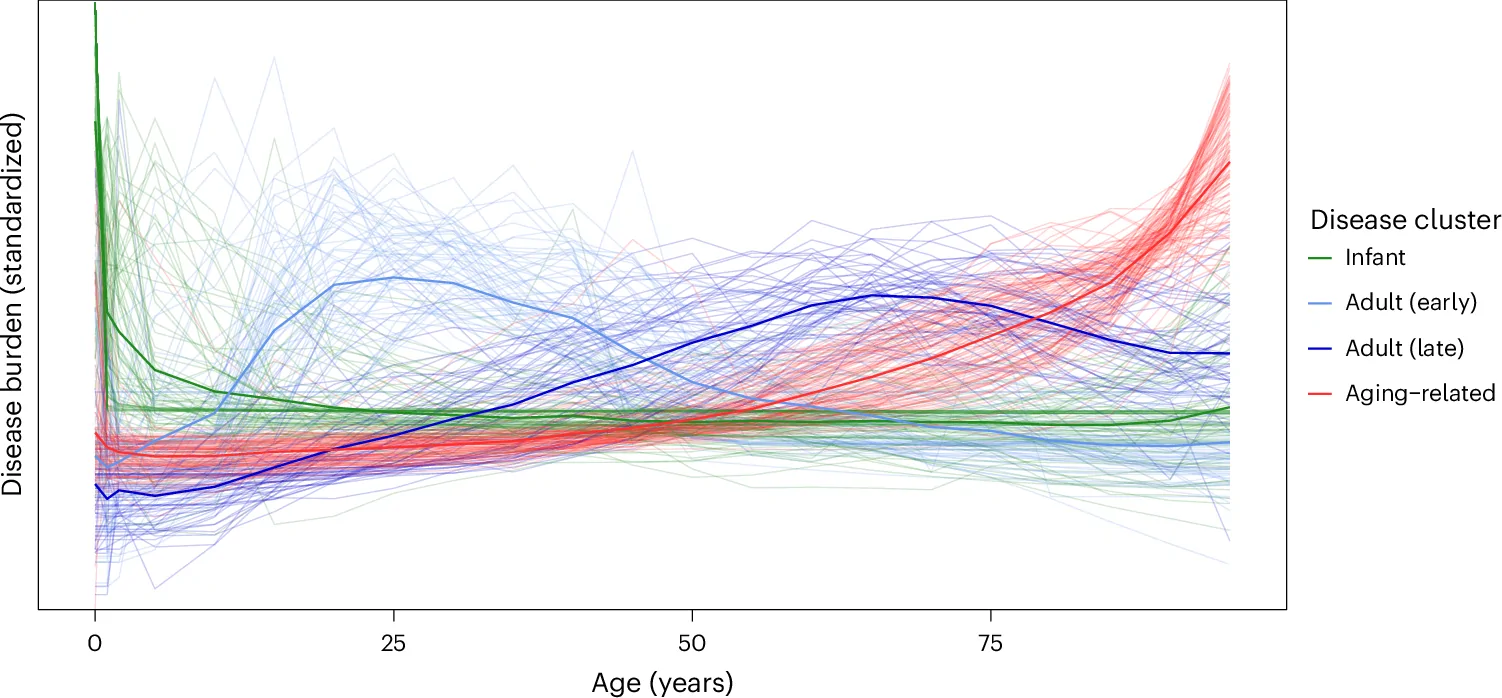
Disease clusters 2023: centroids and individual diseases. Global DALY rates of all GBD diseases across the life cycle, with different diseases allocated to different clusters (indicated by color). The thick solid lines indicate the centroid of each cluster, and fainter lines indicate the individual diseases. DALY rates are for the global population, and diseases are defined at the most detailed level possible. All rates are standardized to have zero mean and unit variance. Source data

The *K*-means++ algorithm suggests four clusters provide an adequate and easily interpretable classification of diseases (Methods). Figure 1 shows the standardized burden for each cluster by age. The clustering algorithm merely identifies diseases with broadly similar life-cycle shapes, rather than using any prespecified criteria. On the basis of Fig. 1, interpreting these clusters is straightforward, and so we name them ‘infant’, ‘adult (early)’, ‘adult (late)’ and ‘aging-related’. The statistically defined timing of peak burden over an individual’s life drives our use of this terminology. The first three are based on the stage of life at which their disease burden peaks. Given our life-cycle approach to classification, all diseases are in some sense age-related, but we use the phrase ‘aging-related’ to define the fourth cluster on the basis of the property that its disease burden rises broadly monotonically with age. Our approach is agnostic regarding whether diseases in a cluster share any related etiology.

The algorithm classifies coronavirus disease (COVID) as an aging-related disease on the basis of its age-related prevalence pattern. In 2023, the prevalence of COVID was too small to make any difference in our results. Whether we include or exclude COVID from the aging-related cluster, our main quantitative and qualitative findings remain unchanged (Supplementary Information).

#### Classification

Infant diseases cover, among others, measles, tetanus, pertussis, malaria, syphilis, respiratory infections and drowning. Early-adult diseases include human immunodeficiency virus/acquired immunodeficiency syndrome and other sexually transmitted diseases, various causes of maternal mortality and physical violence. Late adult diseases include chronic liver disease, mental health problems, some cancers (ovarian, liver and cervical) and some neurodegenerative diseases (motor neuron and multiple sclerosis). Included among aging-related diseases are ischemic heart disease and multiple other cardiovascular conditions, chronic obstructive pulmonary disease, chronic kidney disease, diabetes type 2, some cancers (kidney, liver and prostate), Parkinson’s and dementia. Supplementary Table 3 shows our full classification of GBD categories across clusters and provides information on the robustness of this classification over time.

In terms of number of diseases our aging-related cluster consists mainly of noncommunicable diseases (for 2023, 91% by number and 90% by disease burden). However, while aging-related diseases are mainly noncommunicable, noncommunicable diseases are not mainly aging-related. In total, 48% of noncommunicable diseases are classified as aging-related, 21% as late adult, 16% as early adult and 15% as infant. In particular, several cancers (for example, cervical, ovarian, brain and Hodgkin lymphoma), motor neuron disease and multiple sclerosis are classified as ‘late adult’ given that their burden peaks late in life and then declines.

Related approaches to detecting aging-related diseases include diseases with an incidence that rises quadratically with age^9^, clustering within noncommunicable diseases^10^, using electronic health records to define nine clusters for 278 high-burden diseases (four of which rise continuously with age but with different starting points)^11^ and a study that uses the UK Biobank to identify four aging-related disease clusters with shared molecular etiology^12^. Influenced by our purely epidemiological approach and our focus on a high-level epidemiological transition, we rely instead on a single broad aging-related category defined by a tendency to increase with age.

### Disease burdens

**Fig. 2:**
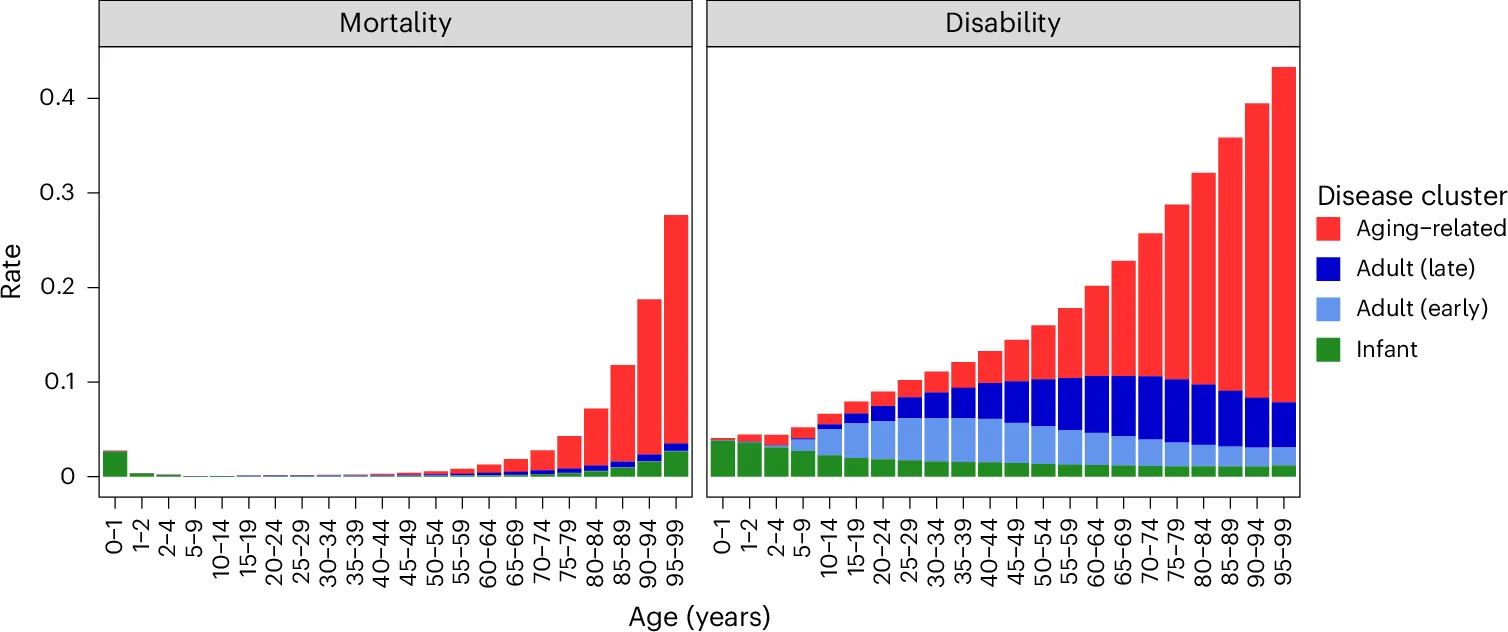
Mortality and disability burden by cluster, 2023. Mortality rates (left) and disability rates (right) for disease clusters calculated using the GBD. These rates are taken directly from the global age-specific death and YLD rates for each cause as provided by the United Nations^1^ and expressed as the rate of deaths/YLDs per person that year due to each cause. The cluster-level rates are the sum of these rates across all causes assigned to that cluster. Source data

Figure 2 shows global mortality and disability rates distributed across clusters by age. The low level of mortality before age 50 years reflects the high level of current global life expectancy, aside from an initial bulge due to infant diseases (mainly in low- and lower-middle-income countries). Adult diseases (early and late) affect disability more than mortality, while aging-related diseases account for most mortality risk. Focusing on disability, the adult diseases (early and late) explain most disability from ages 15 to around 60 years, at which point aging-related diseases dominate.

#### Current burden

Examining the current global disease burden reveals that globally, aging-related diseases are the single most important disease cluster for disability, mortality and the overall disease burden (Supplementary Table 1). Considering World Bank income groups leads to more nuanced results. In terms of years lived with disability (YLDs), aging-related diseases are the main contributors in all regions, including low-income countries. In terms of years of life lost (YLLs), aging-related diseases are the largest cluster in high- and upper-middle-income countries but the second largest in lower-middle- and lower-income countries. For the overall disease burden, aging-related diseases account for the majority in high- and upper-middle-income countries and the second largest (behind infant diseases) in lower-middle- and lower-income countries.

#### Lifetime health burdens

Another way of viewing the health burden is not at a moment in time but over an individual’s life. This is an important distinction for lower- and middle-income countries in the early stages of a demographic transition. These countries have young populations that skew the current disease burden toward infant and early-adult diseases. However, as Table 1 shows, the young can now expect to live a longer life, making their expected lifetime burden different from the country’s current burden.

**Fig. 3:**
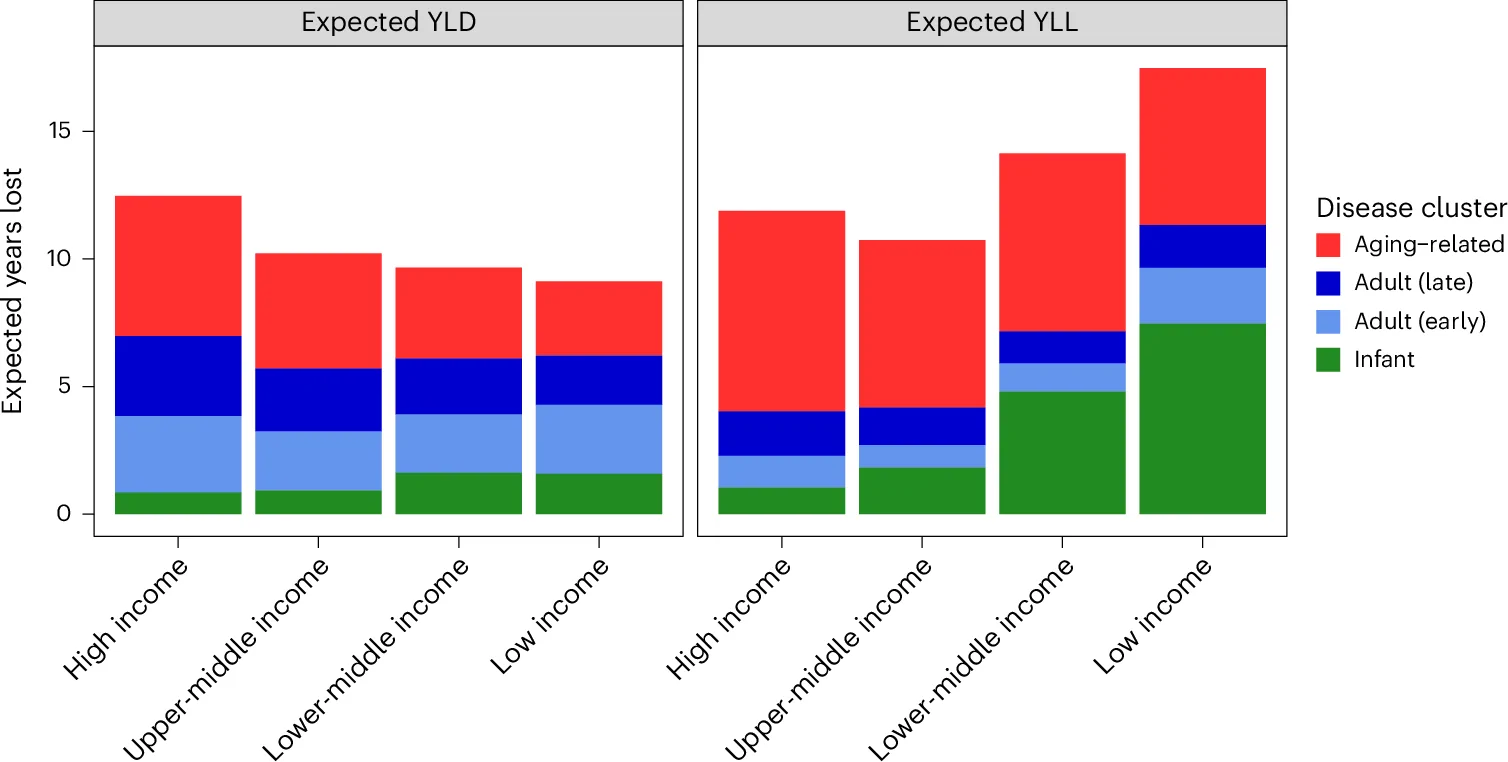
Expected YLDs and YLLs for a newborn in 2023. The expected YLDs (left) and the expected YLLs (right) for a newborn, based on the 2023 YLD and YLL rates for each income group. Source data

To calculate the expected lifetime disease burden, we weight the burden by age and by the probability an individual reaches that age, rather than, as previously, by the number of people of that age (Methods). Figure 3 shows the resulting decomposition of YLDs and YLLs across our income groups and disease clusters.

With the sole exception of low-income countries, the greatest lifetime mortality risk (weighted by remaining years of life) now comes from aging-related diseases. For all income groups, the greatest lifetime disability burden is due to aging-related diseases. In terms of the overall disease burden, aging-related diseases are the single largest contributor to expected lifetime health in every income group. For low-income groups, the total disease burden from aging-related diseases is only narrowly larger than for infant diseases (9.05 versus 9.03), but for the other groups, aging-related disease dominance is much greater. Tackling aging is now the greatest lifetime health challenge for a newborn anywhere in the world.

#### Disease burden transitions by income group

An advantage of our life-cycle perspective to classifying the disease burden is it enables the dynamics of an epidemiological transition to be integrated with those of a demographic transition. Supplementary Table 2 shows for our income groups how the mix of the disease burden among clusters has changed between 1990 and 2023 and how it may change in the future. For future projections, we use mortality and disability rates by income and age band from GBD and then use UN fertility projections by age bands to calculate the future population and therefore disease burden. We provide two versions: one where we assume countries do not benefit from income growth and so stay in their current income bands and one where we assume countries grow. To permit growth transitions, we extrapolate growth in gross domestic product assuming each country grows at the historic rate of its current income group. When a country reaches the next income bracket its growth changes to the average associated with the new band^13^. When a country ascends into the next income bracket, we assume its mortality and disability rates change to those associated with the new income group. These projections are not intended as forecasts, but aim to trace out the implications of expected demographic change (as opposed to other relevant factors such as future pandemics, wars and new treatments, etc.).

Supplementary Table 2 shows that between 1990 and 2023, a major epidemiological shift occurred globally. Infant diseases fell from slightly more than 50% to just less than 30%. Most of the shift occurred through a transition to aging-related diseases, from around one-quarter to just more than two-fifths, and with smaller increases in adult (early) and adult (late). Every single income region experienced an increase in the aging-related burden and a decline in infant diseases, with the biggest shift occurring in middle-income countries and the smallest increase in high-income regions. In other words, a process of convergence is occurring, with other regions catching up with high-income nations in the prevalence of aging-related diseases.

Looking forward, on the basis of changing demographics, this convergence process is projected to continue with the largest increases in aging-related diseases coming from low- and lower-middle-income countries, while high- and higher-middle-income countries will see the share of aging-related diseases converge to around 60%. Allowing for economic growth only speeds up this convergence.

Using our clustering analysis, another perspective on the epidemiological transition emerges. Infant mortality declines and the disease burden shifts mainly to aging-related diseases, with relatively small increases in adult diseases (both early and late). Aging-related diseases are already the largest part of the global disease burden and are projected to rise the fastest in the decades ahead, and to rise fastest of all in lower-middle- and low-income regions. Given that not all chronic diseases are aging-related, but several are adult (late), our approach provides a more nuanced perspective on an epidemiological transition. Being aging-related rather than chronic is most important and defines an epidemiological transition.

### Gains to health from reduced disease prevalence

We can further explore the interaction between the epidemiological and demographic transitions by analyzing the implications of reducing the prevalence of disease clusters for the health of the world’s population. Reductions in disease prevalence change the health of the world’s population for two reasons: (1) health improves at each age for those who are alive and (2) life expectancy improvements mean more people are alive in the future. We statistically decompose improvements in health into these two categories and their interaction.

To do so, we construct a measure, *W*(*t*), of world health (Methods). We define health for everyone at age *a* as 1 minus the sum of YLDs lost to all diseases at that age and then multiply this by the number of people alive at that age before summing across all ages to arrive at a population measure. The GBD dataset does not have YLDs and YLLs for each age, so we use 5-year intervals, except for the first 5 years where we have *<*1, 1 to less than 2 and 2−4-year brackets. We calculate a benchmark for future *W*(*t*) (out to 2125) using GBD mortality, current United Nations population estimates and projected fertility rates.

#### Importance of aging-related diseases

We consider three scenarios of 25%, 50% and 100% reductions in the prevalence of disease clusters. These scenarios are not chosen to be realistic possible outcomes but to illustrate the key dynamic of our reframed epidemiological transition.

If all infant diseases (as measured by our algorithm) were completely eliminated (100% prevalence reduction), global life expectancy would increase by 3.3 years; for early-adult diseases the increase is 0.8 years, and for older-adult diseases the increase is 1.2 years, but for aging-related diseases the increase is 17.4 years (giving a global life expectancy of more than 90 years). These findings reinforce past results that further substantive gains in life expectancy require reducing later-life mortality^14,15^, and, as Fig. 2 shows, that is solely about aging-related diseases.

These calculations also reveal that even total eradication of aging-related diseases does not lead to extreme average lifespans. That is because our disease clusters are identified by when their burden peaks, but they can still have substantial prevalence at other ages. For instance, our analysis shows that older people remain vulnerable to infant diseases, such as intestinal infections, infectious diseases, respiratory infections or animal contact. This nonzero mortality risk from other disease clusters rules out radical life extension in population averages from eradication of aging-related diseases.

Aging-related diseases also have the most powerful impact on healthy life expectancy. Table 1 shows that the ratio of global healthy life expectancy to life expectancy is currently 0.853. Eliminating infant and early adult diseases increases this to 0.91; for older-adult diseases and aging-related diseases, this ratio increases to 0.89 and 0.91, respectively. In the extreme and unrealistic case of complete elimination of aging-related diseases, life expectancy increases by 17.4 years and healthy life expectancy by 20.4 years. Further, only in the case of aging-related diseases does the improvement reflect a reduction of morbidity^16,17^ by reducing the time spent at the end of life in poor health.

**Fig. 4:**
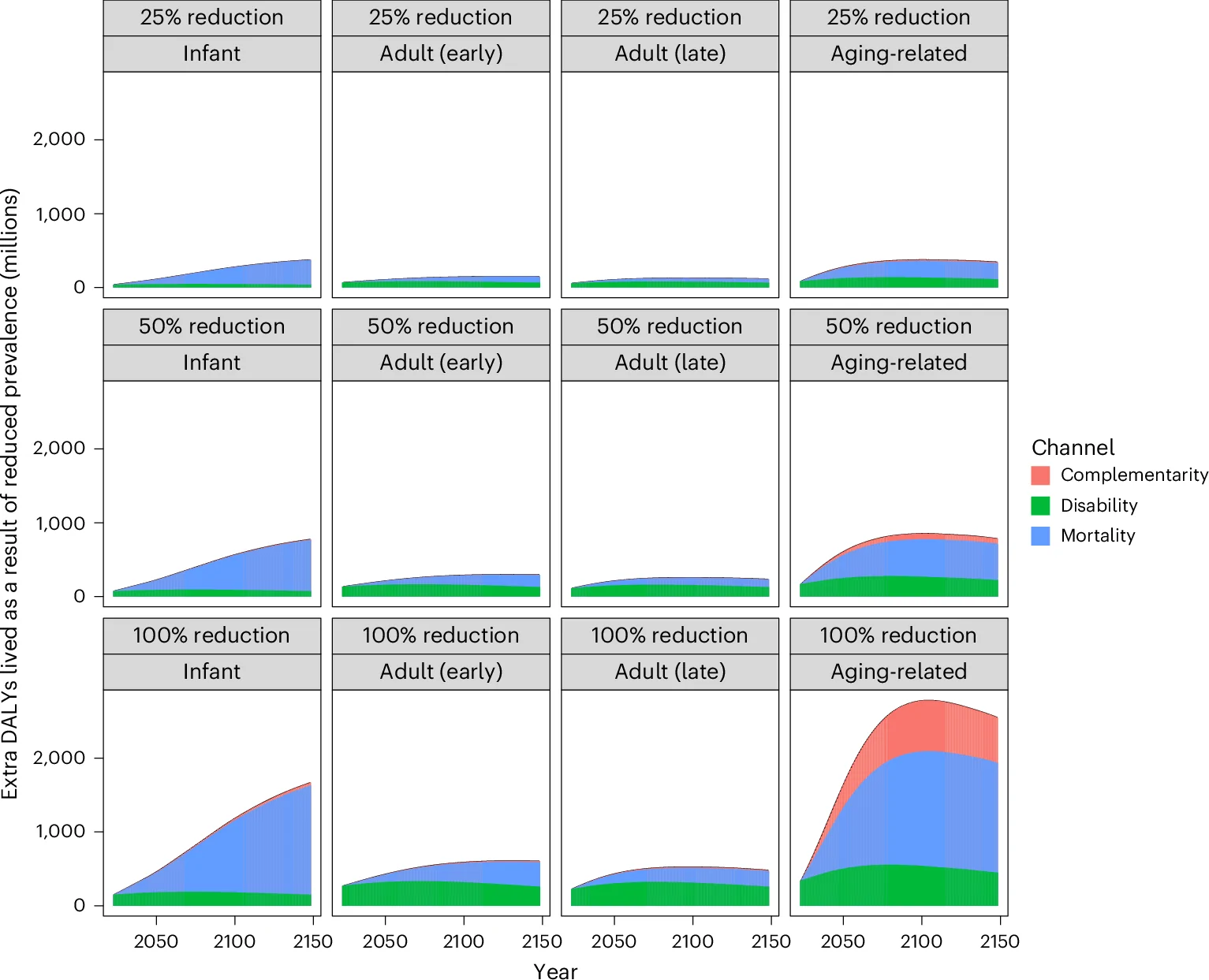
Global health gains from reducing disease prevalence. Increases in DALYs summed across the world population at each moment in time that result from 25%, 50% and 100% permanent reductions in disease prevalence of each cluster. Each row of panels corresponds to the percentage reduction in disease prevalence, while each column of panels corresponds to the disease cluster. Source data

Figure 4 shows the impact over time in *W*(*t*) from reductions in disease prevalence. The gains resulting from eliminating aging-related diseases are the largest across all scenarios and accumulate fastest. For instance, while the initial increase in *W*(*t*) from eliminating aging-related diseases is 50% larger than for eliminating late adult diseases (27% for early adult and 126% for infant), in one 100 years’ time these are 402%, 384% and 320%, respectively.

The importance of the aging-related cluster compared with other clusters is because of:

1. Shifts in the age distribution: The UN fertility projections imply a rising proportion of older people, even without any changes in mortality, so the impact of tackling aging-related diseases increases over time. Reductions in aging-related diseases also leads to more older people living longer, further boosting this effect.
2. Shifts in the disease burden: Aging-related diseases now account for most the most prevalent global diseases, making the aging-related disease category a sum of multiple large disease categories.
3. Complementarity between morbidity and mortality: *W*(*t*) is the sum across all ages of the product of health at age *a* and the number of people alive at that age. It increases because of improvements in health, increases in the population of a given age and a multiplicative effect between gains in health and increases in the number of people. The greater the positive correlation (‘complementarity’) is between improvements in health and improvements in mortality, the larger this effect is. Figure 4 shows that this complementarity is large only for aging-related diseases. As Fig. 2 suggests, aging-related diseases display a high correlation between the timing of mortality (YLL) and morbidity (YLD), whereas non-aging-related diseases tend to either kill you or impair your health. For the infant disease cluster, the correlation between mortality and disability by age is 0.04, for the early-adult cluster it is −0.009, for the late-adult cluster it is −0.03, but for aging-related diseases it is 0.50. The consequence is that, in terms of DALYs gained, aging-related diseases are more than the sum of their parts (the increases in YLLs and YLDs), as they are boosted by a multiplicative effect between YLLs and YLDs.
4. Comorbidities and competing risks: This complementarity in aging-related diseases operates at two levels: at the individual disease level and across diseases within a cluster because of competing risks. The correlation between mortality and morbidity for each individual disease is highest for the aging-related cluster (0.59 as opposed to 0.33, 0.26 and 0.48 for infant, early-adult and older-adult clusters). But aging-related diseases are characterized by greater comorbidities, that is, a high correlation across diseases within the cluster. In other words, aging-related diseases have the highest level of competing risk. The result is that when we aggregate across individual diseases in a cluster the complementarity is maintained for aging-related diseases but reduced for other categories owing to weaker within-group correlation.

#### Increasing returns from tackling aging-related disease

Notably, Fig. 4 shows how, for aging-related diseases, the magnitude of proportional gains to *W*(*t*) from reduced prevalence increases faster than the improvements in prevalence themselves. In other words, the returns are increasing. For non-aging-related diseases, the results of further prevalence reductions are close to linear; that is, the impact of a 50% reduction in prevalence is approximately twice that of a 25% reduction. However, for aging-related diseases the 50% reduction is around 2.2× that of the 25% reduction and the 100% reduction is around 5.9× larger by 2050. By 2100 these ratios have increased to 3.2 and 10.7. Uniquely among our disease clusters, the gains from tackling aging-related diseases become greater the more progress is made in tackling them. Tackling aging-related diseases leads to a virtuous circle such that the more progress is made, the more valuable further progress becomes.

**Fig. 5:**
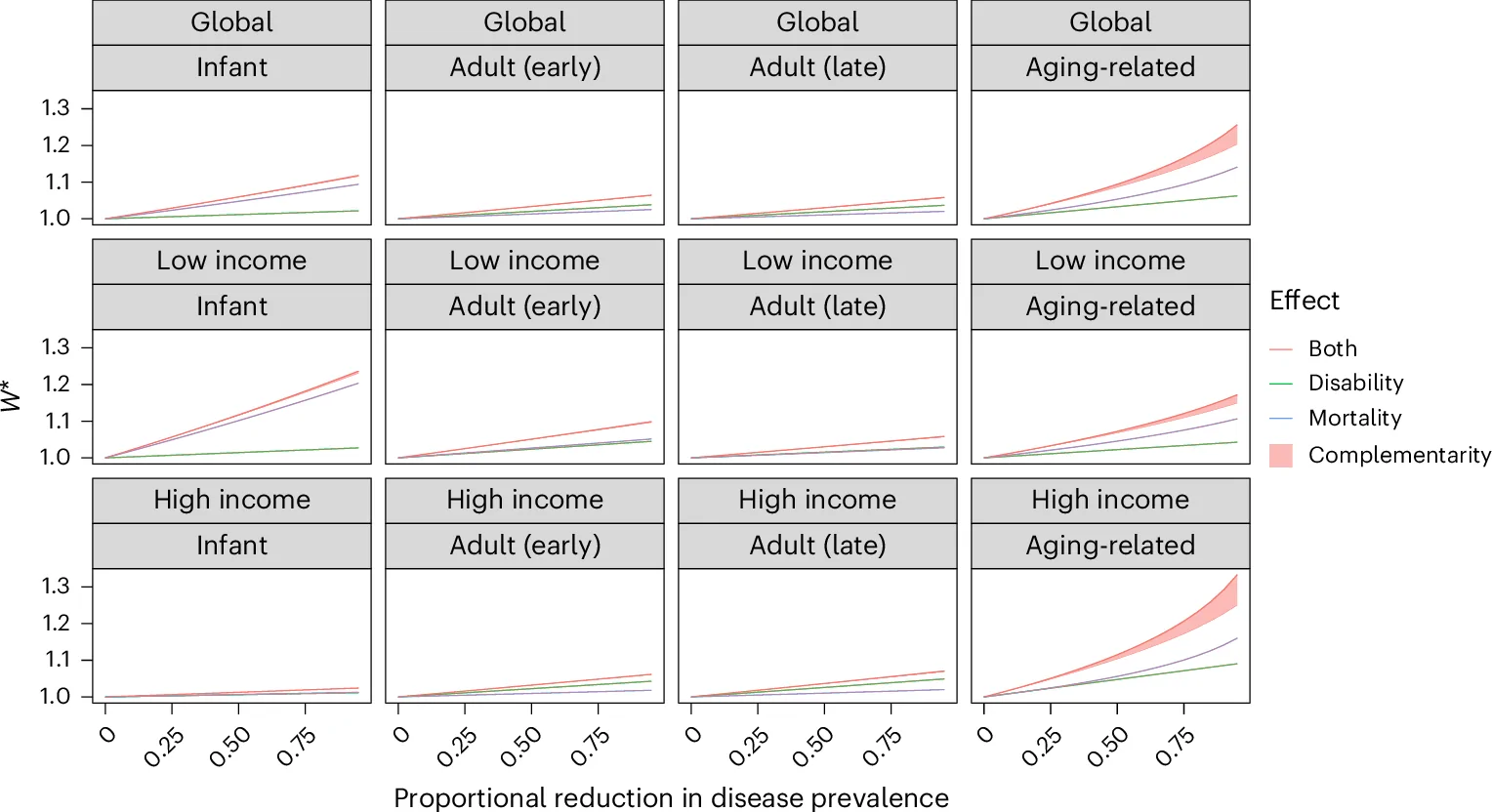
Increasing returns from reducing disease prevalence. The effect of reducing disease prevalence on the level of W*. Each column corresponds to a disease cluster, and the rows to a global, low-income and high-income scope. Lines show the mortality channel, the disability channel and their total; the shaded band represents the complementarity between the mortality and disability channels (the difference between the total effect and the sum of the two channels) and is a deterministic decomposition rather than a statistical uncertainty interval. Source data

To investigate the mechanism behind these increasing returns, we construct a measure W*, which is the sum of *W*(*t*) over the next 125 years. Figure 5 shows how W* varies as the prevalence of each disease cluster changes. We normalize the benchmark to 1 so that a value of 1.1 represents a 10% increase in total future *W*(*t*) relative to the case of no change in prevalence. We show mortality gains (YLLs) in blue, health benefits (YLDs) in green and the total effect in red, while the shaded areas represent the previously discussed complementarity.

Figure 5 shows increasing returns arising through the mortality channel, that is, the increase in population size resulting from higher life expectancy, while the disability channel is linear. Increasing returns arise because reductions in the prevalence of aging-related diseases further increase life expectancy, leading to even more older people who benefit most from further reductions in the prevalence of these diseases.

The size of these increasing returns depends on three factors (derived mathematically in the Methods). The first is a mortality effect: the greater the impact on mortality from reducing disease prevalence the greater are the increasing returns and the bigger the gains to life expectancy and the larger the increase in population size. This effect is largest for aging-related diseases because mortality rates are highest at older ages and so offer the greatest potential for reductions. The second and third factors are the proportional changes in remaining life expectancy and survival rates induced by reductions in disease prevalence.

Aging-related diseases affect mainly the old, who have the lowest remaining life expectancy, so reducing mortality at these ages has the largest proportional effect on remaining life expectancy. Eradicating all infant diseases only increases remaining life expectancy at 70 years by between 2.5% and 13.6% across World Bank income groups. The proportions are even lower for early- and late-adult diseases. Eradicating infant diseases also has relatively small effects on remaining life expectancy at birth. But eradicating aging-related diseases leads to a 125% increase in remaining global life expectancy at age 70 years (111% in high-income countries, 135% in upper-middle-income countries, 124% in lower-middle-income countries and 103% in low-income countries).

These increasing returns point to a different view of the epidemiological transition. When infant mortality was the greatest global disease burden, tackling it led to major welfare gains. But the more infant mortality is reduced, the more welfare gains shift to other parts of the life cycle. A similar argument applies to early- and late-adult diseases. However, aging-related diseases are characterized by increasing prevalence with age. Progress in how we age simply shifts this rising profile out to further years that are now more likely to be experienced^18^. When global life expectancy exceeds 70 years, aging-related diseases are the most important health challenge. The same holds but even more so when global life expectancy exceeds 80 years.

## Discussion

Our analysis shows that aging-related diseases are the most important component of the current global disease burden and represent the greatest lifetime disease burden for a child born in any income group.

Ours is a purely statistical analysis, so noting potential limitations to our analysis is important. First, our identification of aging-related diseases is on the basis of a disease burden that increases with age. This does not imply a single common biological process of aging or that any of the implied underlying biological processes are malleable. Second, in the face of further life expectancy gains, some diseases identified as aging-related may see their burden decline with age, meaning they are not truly ‘aging-related’. This would reduce the importance of this cluster in terms of our quantitative analysis but not undermine the qualitative point we make. Ultimately, if we make progress against all forms of disease, the diseases whose prevalence increases with age become the most important health challenge and the only ones that display increasing returns.

Because our statistical approach to defining aging-related diseases is completely agnostic about biology, it also ignores potential linkages across clusters. For instance, if non-aging-related infectious diseases accelerate aging-related diseases, then the case for focusing on infant diseases or pandemics would be stronger. However, that case becomes stronger because of its link to aging-related diseases.

Further, our finding of increasing returns is based on the use of DALYs as a measure of welfare. This does not account for the economic consequences of longer lives and assumes that the disability weights used in DALYs adequately capture all quality-of-life issues. Allowing for these factors could increase^19^ or diminish our findings of increasing returns.

Our findings have implications for health systems, policies and institutions. The first is that a focus on aging-related diseases does not mean a focus on older people. Because aging-related diseases rise with age, they develop in a compounding manner^20,21^. The challenge is to identify the key periods that influence how we age, which may be in utero, early childhood, adolescence, middle age, older age or all of the above. Further, this life-course perspective reveals that tackling aging-related diseases is a global imperative and not simply an issue for those high-income countries that currently have a high proportion of older people. When global life expectancy exceeds 70 years, how we age is a crucial health challenge regardless of the median age of the population. This emphasizes the importance of tackling aging-related diseases and the growing interest in the biology of aging^19^, and our finding of increasing returns to tackling aging-related diseases implies this is only likely to increase.

Our analysis suggests that as life expectancy increases, an inevitable epidemiological transition toward aging-related diseases occurs. The current health system is built on the considerable historical success of treating disease to raise life expectancy. The future focus needs to be on changing how we age by tackling aging-related diseases. Our results suggest that this is the only way to achieve a compression of morbidity, and the unique complementarity between mortality and morbidity that characterizes aging-related diseases points to the importance of targeting healthy life expectancy rather than life expectancy.

Peter Medawar remarked that aging could only be achieved ‘by the most unnatural experiment of prolonging an animal’s life by sheltering it from the hazards of its ordinary existence’^22^. This is effectively what has happened, given past progress in reducing the prevalence of infant and adult diseases, such that global life expectancy now exceeds 70 years. Our recasting of the epidemiological transition suggests that with global life expectancy now greater than 70 years, a first longevity revolution has ended, such that the young can expect to become the old. Now, a second longevity revolution is required that changes how we age by focusing on aging-related diseases.

## Methods

### Data and simulations

GBD estimates of deaths, DALYs, YLLs and YLDs by cause, age and location were obtained from the Institute for Health Metrics and Evaluation, expressed as numbers and rates for all. We used all causes, ages (at the most granular level possible) and all countries available in the data. The GBD dataset does not have YLDs and YLLs for each age, so we use 5-year intervals, except for the first 5 years where we have <1, 1 to less than 2 and 2−4 brackets. We do not separate by gender. Life expectancy and survival data were obtained from the United Nations World Population Prospects. World Bank income-group classifications are from the World Bank Development Indicators. Our disease clusters are calculated using the global rates across ages, as described in the ‘Data and classification’ section in the ‘Results’.

The cluster-level mortality rates shown in Fig. 2 are the sum of the age-specific global death rates for all causes in that cluster. The death rates provided by GBD are expressed in terms of deaths per 100,000, which we simply converted into deaths per person by dividing by 100,000. The cluster-level disability rates shown in Fig. 2 are the sum of the age-specific global YLD rates for all causes in that cluster, converted into a per-person value. These cluster-specific mortality and disability rates are then used throughout our simulation exercises and to compute expected lifetime burdens. The current burden of disease shown in Fig. 3 is computed directly from the DALY, YLD and YLL values provided by the GBD study.

To represent healthy life expectancy and life expectancy consistently across our simulations, we computed these directly from mortality and disability rates. Let $\mu \left(a\right)$ be the mortality rate at age a and d(a) be the disability rate at age a, both defined as in the previous paragraph. From these mortality rates we can compute survival rates (that is, the probability of a newborn surviving to age a) as

1 $$S\left(a\right)=\mathop{\prod }\limits_{\alpha =0}^{\alpha =a}\left(1-{\mu }_{a}\right).$$

Life expectancy at birth is then calculated as the sum of these survival rates over all ages. Healthy life expectancy at birth then weights each life year by one minus the disability rate. On the basis of current prevalence rates Supplementary Fig. 1 shows the current global burden of disease broken down by clusters and income groups.

Population data are drawn from the United Nations Population Projections 2024 and cover 213 countries. The income group classifications are based on 2023 status, whereby high income includes 86 countries, upper-middle income includes 54 countries, lower-middle income includes 51 countries and low income includes 26 countries. These categories reflect the following percentages of the 2023 global population respectively: 17.3%, 35.0%, 38.1% and 9.1%.

Healthy life expectancy (HLE) is defined by the following, where *d*(*a*) is the disability weight across all diseases at a particular age:

2 $$\mathrm{HLE}\left(0\right)=\mathop{\sum }\limits_{a=0}^{{\rm\infty }}\left(1-d\left(a\right)\right)S\left(a\right).$$

To calculate the probability of death from each type of disease, we take the product of the survival rate and the probability of death at each age from a disease type in each country. Let *µ*_*a,d*_ be the mortality rate from disease *d* at age *a*, while *S*(*a*) is the probability a newborn survives until age *a* based on current mortality rates. The probability a newborn will eventually die of disease *d* is therefore $$\Pr \left(\mathrm{Dies}\,\mathrm{of}\,d\right)$$$$=\mathop{\sum}\nolimits_{{\rm{a}}}S\left(a\right){\mu }_{a,d}$$. To assess the lifetime disease burden, we let *RLE*(*a*) denote remaining life expectancy at age *a*, and define the expected YLL of a newborn from disease *d* as

3 $${{\mathbb{E}}}_{0}\mathrm{YLL}\left(d\right)=\mathop{\sum }\limits_{a}S\left(a\right){\mu }_{a,d}\mathrm{RLE}\left(a\right).$$

To calculate the expected lifetime disability burden we take the probability of a newborn living to a given age and multiply it by the expected disability rate due to the disease at that age. Let *δ*_*a,d*_ be the disability rate due to disease *d* for the average person aged *a*, then the expected YLD for a newborn is

4 $${{\mathbb{E}}}_{0}\mathrm{YLD}=\mathop{\sum }\limits_{a}S\left(a\right){\delta }_{a,d}.$$

Because of future economic growth, the current country groupings are unlikely to remain constant, which we need to permit given the systematic way disease burden varies across these groupings. Therefore, we extrapolate growth in gross domestic product assuming each country grows at the historic rate of its current income group (using the World Bank’s World Development Indicators database from 1960 to 2023, these are 3.0% for low income, 4.3% for lower-middle income and 5.6% for upper-middle income) until they reach the next income bracket, at which point their growth changes to the average associated with the new band^20^. When a country ascends into the next income bracket, we assume its mortality and disability rates change to those associated with the new income group. Given the assumption of positive income growth, all current lower-middle- and upper-middle-income countries will become high income by the end of our simulation period as will around half of the current low-income countries (with the remaining becoming upper-middle income).

### Disease clusters

We use the *K*-means++ algorithm to cluster diseases on the basis of their DALY burden over the life cycle. The DALY burden is first standardized to have zero mean and unit standard deviation. This ensures that we cluster diseases on the basis of the shape of their life-cycle distribution rather than their overall level.

*K*-means++ is a clustering method that automatically groups similar things together in a dataset and is an extension of the *K*-means algorithm. In our case, we are clustering on the basis of the similarity in the DALY–age profile. This works by first choosing several clusters (*k*) and randomly assigning *k* points to function as the initial centers (called centroids) of the clusters. Each data point is then assigned to the closest centroid, after which the centroids are moved to the average position of the points assigned to them. This process is repeated until the centroids converge and clustering is stable.

An issue with the standard *K*-means algorithm is that the seeding of the initial centroids can affect the result. *K*-means++ improves the way the first centroids are chosen. While the first centroid is still chosen randomly, the next one is picked with a preference for being far away from the ones already chosen. This leads to more replicable results and more reliable clustering. We implement the algorithm using the ClusterR R package. Supplementary Table 3 provides a full list of diseases in each cluster.

### Measurement of health and derivation of increasing returns

Define *µ*(*a*), the total mortality for age group *a*, as the sum of mortality rates across all diseases. Analogously, define health *h*(*a*) as 1 minus the sum of YLD rates (*d*(*a*)) across all diseases. Define *ϵ*_*i*_ ≤ 1 as the reduction in prevalence of disease cluster *i*, with *ϵ*_*i*_ < 0 denoting an increase in prevalence, *ϵ*_*i*_ = 1 denoting total eradication and 0 < *ϵ*_*i*_ < 1 denoting reduced prevalence. Mortality and disability are then defined as

5 $$\mu \left(a\right)=\mathop{\sum }\limits_{i}\left(1-{\epsilon }_{i}\right){\mu }_{i}\left(a\right)$$

6 $$h\left(a\right)=1-\mathop{\sum }\limits_{i}\left(1-{\epsilon }_{i}\right){d}_{i}\left(a\right).$$

The number of DALYs lived in the population in period *t* is

7 $$W\left(t\right)=\mathop{\sum }\limits_{a}^{{\rm\infty }}h\left(a,t\right)N\left(a,t\right),$$

where *N*(*a,t*) denotes the number of people aged *a* alive at time *t*. We consider as our measure of welfare the sum of all current and future health-adjusted life years of the world population, that is,

8 $${W}^{* }\left(t\right)=\mathop{\sum }\limits_{t=0}^{T}\mathop{\sum }\limits_{a=0}^{{\rm\infty }}h\left(a,t\right)N\left(a,t\right),$$

where *T* is a fixed future horizon.

We are interested in how this measure reacts to changes in the prevalence of disease clusters. To capture this, we investigate $\frac{\Delta {W}^{* }}{\Delta {{{\epsilon }}}_{{\rm{i}}}}\frac{1}{{W}^{* }}$, namely the semi-elasticity of $W{\left(t\right)}^{* }$ with respect to the eradication of disease *i* (*ϵ*_*i*_), or the percentage change in total welfare resulting from an extra percentage point eradication of disease cluster *i*.

Very small changes in the prevalence of a disease will affect *W*^∗^ through either changes in *h*(*a,t*)—that is, health—or through changes in *N*(*a,t*)—that is, reductions in mortality that keep more people alive and lead to a larger population, as shown in the following first-order approximation (where LS denotes maximum lifespan, which we set to 250, well above the length of life anyone lives to in our simulations):

9 $$\begin{array}{ll}\displaystyle\frac{\Delta {W}^{\,* }}{\Delta {{{\epsilon }}}_{{\rm{i}}}}\displaystyle\frac{1}{{W}^{* }}\approx \displaystyle\frac{1}{{W}^{* }}\left[\mathop{\sum }\limits_{t=0}^{T}\mathop{\sum }\limits_{a=0}^{\rm{LS}}h\left(a,t\right)\displaystyle\frac{\partial N\left(a,t\right)}{\partial \mu \left(.\right)}\displaystyle\frac{\partial \mu \left(.\right)}{\partial {\epsilon }_{i}}\right.\\\left.+\mathop{\sum }\limits_{t=0}^{T}\mathop{\sum }\limits_{a=0}^{\rm{LS}}\displaystyle\frac{\partial h\left(a,t\right)}{\partial {\epsilon }_{i}}N\left(a,t\right)\right]=\displaystyle\frac{1}{{W}^{* }}\left[\displaystyle\frac{\partial {W}^{* }}{\partial \mu }\displaystyle\frac{\partial \mu }{\partial {\epsilon }_{i}}+\displaystyle\frac{\partial {W}^{* }}{\partial h}\displaystyle\frac{\partial h}{\partial {\epsilon }_{i}}\right]\end{array}$$

However, as discussed in the ‘Disease clusters’ section, potential nonlinearities exist, particularly in the case of aging-related diseases. Taking a second-order approximation is therefore useful, namely

10 $$\begin{array}{ll}\displaystyle\frac{{\Delta }{W}^{\,* }}{{W}^{* }}\approx \displaystyle\frac{1}{{W}^{* }}\left[\left(\displaystyle\frac{\partial {W}^{* }}{\partial \mu }\displaystyle\frac{\partial \mu }{\partial {\epsilon }_{i}}+\displaystyle\frac{\partial {W}^{* }}{\partial h}\displaystyle\frac{\partial h}{\partial {\epsilon }_{i}}\right){\Delta }{\epsilon }_{i}\right.\\\left.+\displaystyle\frac{1}{2}\left(\displaystyle\frac{{\partial }^{2}{W}^{* }}{\partial {\mu }^{2}}\displaystyle\frac{{\partial }^{2}\mu }{\partial {\epsilon }_{i}^{2}}+2\displaystyle\frac{{\partial }^{2}{W}^{* }}{\partial \mu \partial h}\displaystyle\frac{\partial \mu \partial h}{\partial {\epsilon }_{i}^{2}}+\displaystyle\frac{{\partial }^{2}{W}^{* }}{\partial {h}^{2}}\displaystyle\frac{{\partial }^{2}h}{\partial {\epsilon }_{i}^{2}}\right){{\Delta }\epsilon }_{i}^{2}\right].\end{array}$$

This second-order approximation shows that our measure of welfare (W*) responds to changes in disease prevalence through the following channels:

1. $\frac{\partial {W}^{\,* }}{\partial \mu }\frac{\partial \mu }{\partial {\epsilon }_{i}}$: the direct impact on health arising from reductions in mortality and subsequent increases in the population.
2. $\frac{\partial {W}^{\,* }}{\partial h}\frac{\partial h}{\partial {\epsilon }_{i}}$: the direct impact of improvements in health from lower disease prevalence.
3. $\frac{{\partial }^{2}{W}^{\,* }}{\partial {\mu }^{2}}\frac{{\partial }^{2}\mu }{\partial {\epsilon }_{i}^{2}}$: potential increasing returns to mortality prevention.
4. $\frac{{\partial }^{2}{W}^{\,* }}{\partial {h}^{2}}\frac{{\partial }^{2}h}{\partial {\epsilon }_{i}^{2}}$: potential increasing returns from improvements in health.
5. $\frac{{\partial }^{2}{W}^{* }}{\partial \mu \partial h}\frac{\partial \mu \partial h}{\partial {\epsilon }_{i}^{2}}$: the complementarity between mortality and disability gains.

Channels 1 and 2 refer to first-order effects, while channel 4 turns out to be zero because *W*^∗^ is linear in *h*. A combination of channels 3 and 5 drives the increasing returns observed in the ‘Disease clusters’ section. Channel 5 is straightforward, reflecting the complementarity between mortality and disability that is pronounced in aging-related diseases and represented by the shaded red area in Fig. 5.

Channel 3 captures increasing returns to reducing mortality from aging-related diseases and works through *N*(*a,t*), the population distribution. Assuming constant fertility, the steady-state population distribution is the integral of the survival curve, which in turn is simply life expectancy at birth, that is,

11 $${\rm{LE}}\left(0\right)={\int }_{0}^{\infty }S\left(a\right){da}={\int }_{0}^{\infty }\exp \left(-{\int }_{0}^{\infty }\mu \left(x\right){dx}\right){da}$$

Changes in life expectancy (LE) in response to a variable *ϵ*_*i*_ that changes mortality can be calculated^23^ by summing up the changes in mortality over all ages, weighted by the remaining person-years of expected life at each age

12 $$\frac{\mathrm{dLE}\left(0\right)}{{\rm{d}}{\epsilon }_{i}}=-{\int }_{0}^{{\rm\infty }}\frac{\partial \mu \left(a\right)}{\partial {\epsilon }_{i}}\mathrm{LE}\left(a\right)S\left(a\right){da}.$$

This has three components:

1. Mortality, $\frac{\partial \mu \left(a\right)}{\partial {\epsilon }_{i}}$. This is straightforward: if *ϵ*_*i*_ has a bigger effect on mortality it has a bigger effect on life expectancy.
2. Remaining life expectancy, LE(*a*). This is forward looking and reflects mortality rates after *a*, so that reducing mortality is more valuable at ages where a lot of life remains.
3. Survival, *S*(*a*). This is backward looking and reflects mortality rates before age *a*, so that reducing mortality is more valuable at ages that a newborn is more likely to experience in the future.

To investigate the mechanism behind increasing returns we need to examine the second-order term:

13 $$\begin{array}{l}\displaystyle\frac{{{\rm{d}}}^{2}\mathrm{LE}\left(0\right)}{{\rm{d}}{\epsilon }_{i}^{2}}=-{\displaystyle\int }_{0}^{\infty }\left[\displaystyle\frac{{\partial }^{2}\mu \left(a\right)}{\partial {\epsilon }_{i}^{2}}\mathrm{LE}\left(a\right)S\left(a\right)\right.\\\left.+\displaystyle\frac{\partial \mu \left(a\right)}{\partial {\epsilon }_{i}}S\left(a\right)\displaystyle\frac{\partial {\rm{LE}}\left(a\right)}{\partial {\epsilon }_{i}}+\displaystyle\frac{\partial \mu \left(a\right)}{\partial {\epsilon }_{i}}\mathrm{LE}\left(a\right)\displaystyle\frac{\partial S\left(a\right)}{\partial {\epsilon }_{i}}\right]{da}.\end{array}$$

The more positive this second derivative is, the stronger the increasing returns. Note that $\frac{{\partial }^{2}\mu (a)}{\partial {\epsilon }_{i}^{2}}=0$ in our case because we define disease prevalence reduction in percentage terms relative to the current burden. Then, noting that $\frac{\partial y}{\partial x}=y\frac{\partial \log y}{\partial x}$, we can rewrite this as

14 $$\frac{{d}^{2}{\rm{LE}}\left(0\right)}{d{\epsilon }_{i}^{2}}=-{\int }_{0}^{\infty }\frac{\partial \mu \left(a\right)}{\partial {\epsilon }_{i}}S\left(a\right){\rm{LE}}(a)\left[\frac{\partial \log {\rm{LE}}(a)}{\partial {\epsilon }_{i}}+\frac{\partial \log S(a)}{\partial {\epsilon }_{i}}\right]{da}$$

which gives an intuitive explanation for why eradicating aging-related diseases has increasing returns for life expectancy and our *W*^∗^(*t*) term. At the ages where they have the highest mortality effect, aging-related diseases have a greater proportional effect on both remaining life expectancy (LE(*a*)) and survival until that age (*S*(*a*)) than is the case for other diseases. Infant diseases provide a useful contrast here: infant diseases are proportionally a very small part of LE(*a*) and *S*(*a*) at the ages where they have their largest mortality effect. This in turn is a direct result of the fact that aging-related diseases increase with age, which always makes the mortality effect largest where the proportional effects on LE and *S*(*a*) are greatest. For every other disease cluster, these terms reduce in importance as more progress is made.

### Ethics statement

This study used publicly available, de-identified data and did not involve the collection of new human data or interaction with human participants. Therefore, institutional review board approval and informed consent were not required.

### Reporting summary

Further information on research design is available in the Nature Portfolio Reporting Summary linked to this article.

## Supplementary information

Supplementary Information Additional data and results, Supplementary Tables 1–4 and Supplementary Figs. 1 and 2 .

Reporting Summary

## Source data

Source Data Figs. 1–5 Source data files for Figs. 1–5.

## Data Availability

This study analyzes publicly available, aggregated datasets and does not generate new data. GBD estimates of deaths, DALYs, YLLs and YLDs by cause, age and location were obtained from the Institute for Health Metrics and Evaluation Global Health Data Exchange at https://vizhub.healthdata.org/gbd-results/. Life expectancy and survival data were obtained from the United Nations World Population Prospects at https://population.un.org/wpp/. World Bank income-group classifications are available at https://datahelpdesk.worldbank.org/knowledgebase/articles/906519. All datasets are freely available from these sources under their respective terms of use. The processed data underlying the figures and tables are available in the replication package available via GitHub at https://github.com/julianashwin/replication-package-reframing-epidemiological-transition.

## Code availability

All code is in the replication package available via GitHub at https://github.com/julianashwin/replication-package-reframing-epidemiological-transition. Analysis was done in the programming language R and the following packages were used: readxl, tidyverse, janitor, beepr, foreach, doParallel, iterators, stringr, ClusterR, stargazer, gt, factoextra, scales, ggplot2 and ggpubr.

## References

[1] World Population Prospects. *United Nations* https://population.un.org/wpp (2024).

[2] Sanderson, W. and Scherbov, S. *Prospective Longevity: A New Vision of Population Aging* (Harvard Univ. Press, 2019).

[3] Omran, A. The epidemiologic transition: a theory of the epidemiology of population change. *Millbank Q.* **39**, 509–538 (1971). 5155251

[4] Kennedy, B. et al. Geroscience: linking aging to chronic disease. *Cell* **159**, 709–713 (2014). 25417146 10.1016/j.cell.2014.10.039 PMC4852871

[5] López-Otın, C., Blasco, M., Partridge, L., Serrano, M. & Kroemer, G. The hallmarks of aging. *Cell* **153**, 1194–1217 (2013). 23746838 10.1016/j.cell.2013.05.039 PMC3836174

[6] López-Otın, C., Blasco, M., Partridge, L., Serrano, M. & Kroemer, G. The hallmarks of aging: an expanding universe. *Cell* **186**, 243–278 (2023). 36599349 10.1016/j.cell.2022.11.001

[7] Arthur, D. & Vassilivitskii, S. *k*-Means++: the advantages of careful seeding. In *Proc. Eighteenth Annual ACM-SIAM Symposium on Discrete Algorithms* 1027–1035 (Society for Industrial and Applied Mathematics, 2007).

[8] *Global Burden of Disease Study* (Institute for Health Metrics and Evaluation, Global Burden of Disease Collaborative Network, 2024). 10.1093/ije/dyae122 PMC11427672 39327917

[9] Chang, A., Skirbekk, V., Tyrolvolas, S., Kassebaum, N. & Dieleman, J. Measuring population ageing: an analysis of the Global Burden of Disease study 2017. *Lancet Public Health* **4**, e159–e167 (2019). 30851869 10.1016/S2468-2667(19)30019-2 PMC6472541

[10] Le Couteur, D. & Thillainadesan, J. What is an aging-related disease? An epidemiological perspective. *J. Gerontol. A Biol. Sci. Med. Sci.* **77**, 2168–2174 (2022). 35167685 10.1093/gerona/glac039 PMC9678203

[11] Kuan, V. et al. Data-driven identification of ageing-related diseases from electronic health records. *Sci. Rep.* **11**, 938 (2021). 33536532 10.1038/s41598-021-82459-y PMC7859412

[12] Donertas, H., Fabian, D., Valenzuela, M., Partridge, L. & Thornton, J. Common genetic associations between age-related diseases. *Nat. Aging* **1**, 400–412 (2021). 33959723 10.1038/s43587-021-00051-5 PMC7610725

[13] Nandi, A. et al. Global and regional projections of the economic burden of Alzheimer’s disease and related dementias from 2019 to 2050: a value of statistical life approach. *eClin. Med.* **51**, 101580 (2022). 10.1016/j.eclinm.2022.101580 PMC9310134 35898316

[14] Olshansky, S. et al. Implausibility of radical life extension in humans in the twenty-first century. *Nat. Aging* **4**, 1635–1642 (2024). 39375565 10.1038/s43587-024-00702-3 PMC11564081

[15] Ashwin, J. & Scott, A. J. A Bayesian model of later life mortality trends and implications for longevity. *J. R Stat. Soc. Ser. A* **189**, 834–850 10.1093/jrsssa/qnaf026 (2026).

[16] Fries, J. F. Aging, natural death, and the compression of morbidity. *New Engl. J. Med.* **303**, 130–135 (1980). 7383070 10.1056/NEJM198007173030304

[17] Crimmins, E. M. Lifespan and healthspan: past, present, and promise. *Gerontologist* **55**, 901–911 (2015). 26561272 10.1093/geront/gnv130 PMC4861644

[18] Olshansky, S. J. & Ault, A. B. The fourth stage of the epidemiologic transition: the age of delayed degenerative diseases. *Milbank Q.* **64**, 355–391 (1986). 3762504

[19] Scott, A. J., Ellison, M. & Sinclair, D. A. The economic value of targeting aging. *Nat. Aging* **1**, 616–623 (2021). 37117804 10.1038/s43587-021-00080-0 PMC10154220

[20] Bloom, D. E. et al. The economic burden of chronic diseases: estimates and projections for China, Japan, and South Korea. *J. Econ. Ageing* **17**, 100163 (2020).

[21] Schindler, Y. & Scott, A. J. The macroeconomic impact of chronic disease in the United Kingdom. *J. Econ. Ageing* **32**, 100590. (2025).

[22] Medawar, P. B. *An Unsolved Problem in Biology* (Lewis, 1951).

[23] Wrycza, T. F. & Baudisch, A. How life expectancy varies with perturbations in age-specific mortality. *Demogr. Res.* **27**, 365–376 (2012).

