# Supplementary material for: Reframing the epidemiological transition as increasing returns to tackling aging-related diseases
Source: Nat Aging. 2026 Sep 2;6(9):2028–37. doi: 10.1038/s43587-026-01210-2 (PMC13577912; doi:10.1038/s43587-026-01210-2)
Supplement: Supplementary file 1 [file 43587_2026_1210_MOESM1_ESM.pdf]

nature aging

Supplementary information https://doi.org/10.1038/s43587-026-01210-2

Reframing the epidemiological transition as increasing returns to tackling aging-related diseases

In the format provided by the authors and unedited

Supplementary Data File

Additional Data and Results

Supplementary Table 1: Current Global Disease Burden by Cluster, 2023 (percent)

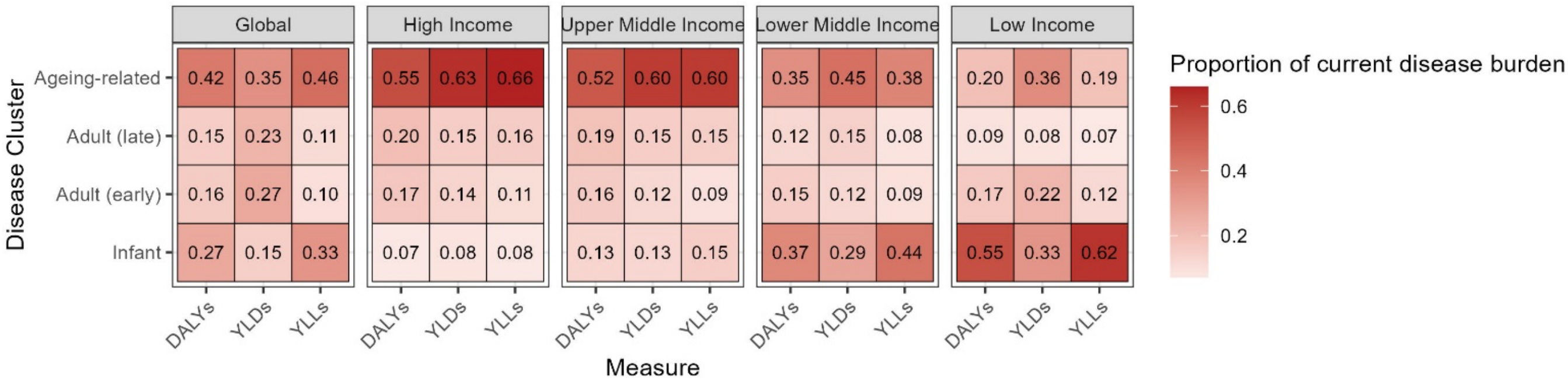

Note: This table shows the percentage breakdown of years lost to disability (YLDs), years of life lost (YLLs), and the total disease burden (YLLs + YLDs = disability-adjusted life years (DALYs)) across four disease clusters and World Bank income groups.

Supplementary Table 2: Epidemiological Transition: Past and Future (Percentage of Disease Burden Attributed to Disease Clusters)

Location	Cluster	1990	2023	2050	2075	2100	2125
**Without economic growth transitions**							
Global	Infant	52.3	29.3	25.4	23.9	23.3	23.2
	Early adult	11	15.6	13.4	12.7	12.4	12.1
	Late adult	10.5	14.8	14.5	14.1	13.9	13.8
	Ageing-related	26.2	40.2	46.7	49.3	50.4	50.9
High income	Infant	12.4	7.4	6.7	6.6	6.7	6.7
	Early adult	16.1	17.2	13.5	12.6	12.9	13.1
	Late adult	20.2	20.2	18.8	18.1	18.1	18.2
	Ageing-related	51.3	55.2	61.0	62.7	62.3	61.9
Upper-middle income	Infant	38.3	13.6	10.4	9.7	9.7	9.8
	Early adult	12.5	16.1	11.3	10.1	10	10.2
	Late adult	14.6	19.1	17.5	16.7	16.3	16.6
	Ageing-related	34.6	51.2	60.7	63.6	64.0	63.4
Lower-middle income	Infant	69.7	38.6	30.7	26.7	24.8	24
	Early adult	8.1	14.8	13.2	11.9	11.3	10.9
	Late adult	5.9	12.1	13	13.1	13.1	13.1
	Ageing-related	16.3	34.6	43.0	48.2	50.7	52.0
Low income	Infant	75.1	58	48.8	40.8	35.4	33.3
	Early adult	11.3	15.4	17.3	17.2	16.2	15.3
	Late adult	4.2	8.3	10.3	11.7	12.4	12.5
	Ageing-related	9.4	18.3	23.6	30.3	35.9	39.0
**Allowing for economic growth transitions**							
Global	Infant	52.3	29.3	16.5	11.8	8.9	7.7
	Early adult	11	15.6	14.9	14.9	14.5	14.6
	Late adult	10.5	14.8	17.4	18	18.3	18.6
	Ageing-related	26.2	40.2	51.2	55.3	58.2	59.1
High income	Infant	12.4	7.4	6.7	6.6	6.7	6.7

	Early adult	16.1	17.2	13.5	12.6	12.9	13.1
	Late adult	20.2	20.2	18.8	18.1	18.1	18.2
	Ageing-related	51.3	55.2	61.0	62.7	62.3	61.9
Upper-middle income	Infant	38.3	13.6	6.9	6.6	6.6	6.7
	Early adult	12.5	16.1	14.7	12.8	12.7	13
	Late adult	14.6	19.1	19.5	18.3	17.9	18.2
	Ageing-related	34.6	51.2	58.8	62.3	62.8	62.1
Lower-middle income	Infant	69.7	38.6	17.3	9	7.6	7.4
	Early adult	8.1	14.8	15.5	16.8	15.7	15
	Late adult	5.9	12.1	17.5	19.3	19	18.8
	Ageing-related	16.3	34.6	49.6	54.9	57.6	58.8
Low income	Infant	75.1	58	43.5	29.7	15.7	9.4
	Early adult	11.3	15.4	14.9	14.9	14.1	15.4
	Late adult	4.2	8.3	11.1	14.1	17.3	18.8
	Ageing-related	9.4	18.3	30.5	41.3	52.9	56.4

Table A3 lists the diseases in each cluster. Column heads define disease clusters, with the total global disease burden attributable to each cluster in parentheses. Rows show individual diseases' percentages of total disease burden in 2023 and in parentheses are the percentages of the individual diseases within each cluster. Noncommunicable diseases are shaded blue, communicable diseases are red, injuries are green, and other is purple.

Supplementary Table 3: Assignment of Diseases to Clusters, Percent of Total Disease Burden in 2023 (Percent of Disease Burden within Cluster)

Infant (26.86%)	Adult early (16.01%)	Adult late (15.30%)	Ageing-related (41.83%)
Lower respiratory infections: 3.529% (13.141%)	Anxiety disorders: 1.98% (12.362%)	Low back pain: 2.518% (16.458%)	Ischemic heart disease: 6.879% (16.444%)
Neonatal preterm birth: 2.51% (9.348%)	Major depressive disorder: 1.699% (10.608%)	Tracheal, bronchus, and lung cancer: 1.668% (10.9%)	Diabetes mellitus type 2: 3.112% (7.439%)
Diarrheal diseases: 2.019% (7.518%)	Migraine: 1.462% (9.127%)	Other musculoskeletal disorders: 1.624% (10.618%)	Intracerebral hemorrhage: 2.796% (6.683%)
Neonatal encephalopathy due to birth asphyxia and trauma: 1.965% (7.315%)	HIV/AIDS resulting in other diseases: 1.22% (7.619%)	Pedestrian road injuries: 0.752% (4.917%)	Chronic obstructive pulmonary disease: 2.675% (6.396%)
Malaria: 1.844% (6.866%)	Self-harm by other specified means: 1.154% (7.206%)	Neck pain: 0.741% (4.842%)	Falls: 2.654% (6.345%)
Drug-susceptible tuberculosis: 1.418% (5.279%)	Motor vehicle road injuries: 1.07% (6.682%)	Esophageal cancer: 0.502% (3.279%)	Ischemic stroke: 2.394% (5.722%)
Dietary iron deficiency: 1.407% (5.237%)	Motorcyclist road injuries: 0.65% (4.057%)	Cervical cancer: 0.474% (3.099%)	Age-related and other hearing loss: 1.898% (4.538%)
Congenital heart anomalies: 0.948% (3.528%)	Alcohol use disorders: 0.645% (4.027%)	Chronic hepatitis B including cirrhosis: 0.467% (3.055%)	Alzheimer's disease and other dementias: 1.421% (3.397%)
Neonatal sepsis and other neonatal infections: 0.811% (3.02%)	Other gynecological diseases: 0.61% (3.807%)	Other unintentional injuries: 0.457% (2.987%)	COVID-19: 1.148% (2.745%)
Other neonatal disorders: 0.625% (2.328%)	Schizophrenia: 0.606% (3.785%)	Osteoarthritis knee: 0.45% (2.941%)	Hypertensive heart disease: 1.015% (2.426%)
Meningitis: 0.625% (2.327%)	Conflict and terrorism: 0.472% (2.949%)	Chronic hepatitis C including cirrhosis: 0.393% (2.571%)	Colon and rectum cancer: 0.935% (2.235%)
Drowning: 0.608% (2.263%)	Opioid use disorders: 0.457% (2.855%)	Cirrhosis due to alcohol: 0.346% (2.261%)	Asthma: 0.924% (2.209%)
Idiopathic epilepsy: 0.551% (2.053%)	Physical violence by firearm: 0.389% (2.427%)	Other exposure to mechanical forces: 0.339% (2.217%)	Breast cancer: 0.88% (2.104%)

Protein-energy malnutrition: 0.503% (1.873%)	HIV/AIDS drug-susceptible tuberculosis: 0.357% (2.228%)	Other mental disorders: 0.338% (2.208%)	Stomach cancer: 0.804% (1.922%)
Measles: 0.446% (1.661%)	Premenstrual syndrome: 0.295% (1.841%)	Brain and central nervous system cancer: 0.328% (2.141%)	Chronic kidney disease due to other and unspecified causes: 0.597% (1.426%)
Physical violence by other means: 0.413% (1.538%)	Bipolar disorder: 0.273% (1.703%)	Dysthymia: 0.304% (1.99%)	Rheumatic heart disease: 0.518% (1.237%)
Fire, heat, and hot substances: 0.381% (1.42%)	Physical violence by sharp object: 0.204% (1.277%)	Periodontal diseases: 0.252% (1.649%)	Pancreatic cancer: 0.439% (1.049%)
Pertussis: 0.362% (1.349%)	Conduct disorder: 0.172% (1.074%)	Ovarian cancer: 0.232% (1.514%)	Other cardiovascular and circulatory diseases: 0.413% (0.987%)
Autism spectrum disorders: 0.351% (1.309%)	Other transport injuries: 0.169% (1.053%)	Lip and oral cavity cancer: 0.231% (1.508%)	Subarachnoid hemorrhage: 0.402% (0.962%)
Other unspecified infectious diseases: 0.331% (1.233%)	Other oral disorders: 0.159% (0.99%)	Gastroesophageal reflux disease: 0.23% (1.504%)	Atopic dermatitis: 0.396% (0.946%)
Other congenital birth defects: 0.275% (1.022%)	Attention-deficit/hyperactivity disorder: 0.133% (0.832%)	Liver cancer due to hepatitis B: 0.215% (1.405%)	Edentulism: 0.36% (0.86%)
Syphilis: 0.244% (0.91%)	Idiopathic developmental intellectual disability: 0.13% (0.814%)	Near vision loss: 0.201% (1.317%)	Chronic kidney disease due to diabetes mellitus type 2: 0.342% (0.818%)
Upper respiratory infections: 0.224% (0.833%)	Maternal hemorrhage: 0.114% (0.711%)	Refraction disorders: 0.185% (1.207%)	Chronic kidney disease due to hypertension: 0.338% (0.808%)
Digestive congenital anomalies: 0.217% (0.809%)	Maternal hypertensive disorders: 0.11% (0.689%)	Cyclist road injuries: 0.176% (1.154%)	Atrial fibrillation and flutter: 0.331% (0.792%)
Invasive nontyphoidal salmonella (iNTS): 0.209% (0.778%)	Bulimia nervosa: 0.107% (0.666%)	Tension-type headache: 0.166% (1.083%)	Other cardiomyopathy: 0.328% (0.785%)
Pulmonary aspiration and foreign body in airway: 0.197% (0.734%)	Self-harm by firearm: 0.106% (0.659%)	Contact dermatitis: 0.134% (0.873%)	Prostate cancer: 0.318% (0.761%)
Typhoid fever: 0.194% (0.721%)	Other direct maternal disorders: 0.089% (0.553%)	Multidrug-resistant tuberculosis without extensive drug resistance: 0.133% (0.868%)	Gallbladder and biliary diseases: 0.292% (0.698%)
Other neglected tropical diseases: 0.184% (0.684%)	Iodine deficiency: 0.08% (0.501%)	Larynx cancer: 0.127% (0.832%)	Parkinson's disease: 0.285% (0.682%)
Urticaria: 0.18% (0.67%)	Caries of permanent teeth: 0.079% (0.491%)	Other pharynx cancer: 0.119% (0.778%)	Other non-Hodgkin lymphoma: 0.284% (0.679%)
Exposure to forces of nature: 0.171% (0.637%)	Schistosomiasis: 0.074% (0.462%)	Chronic kidney disease due to diabetes mellitus type 1: 0.109% (0.715%)	Chronic kidney disease due to glomerulonephritis: 0.264% (0.63%)
Venomous animal contact: 0.171% (0.637%)	Endometriosis: 0.068% (0.426%)	Malignant neoplasm of bone and articular cartilage: 0.108% (0.705%)	Other endocrine, metabolic, blood, and immune disorders: 0.261% (0.624%)
Hemolytic disease and other neonatal jaundice: 0.167% (0.622%)	Viral skin diseases: 0.065% (0.405%)	Uterine cancer: 0.104% (0.678%)	Thyroid diseases: 0.258% (0.617%)
Acute lymphoid leukemia: 0.157% (0.583%)	Amphetamine use disorders: 0.063% (0.392%)	Psoriasis: 0.095% (0.619%)	Paralytic ileus and intestinal obstruction: 0.257% (0.615%)
Sickle cell disorders: 0.154% (0.573%)	Maternal sepsis and other maternal infections: 0.06% (0.373%)	Nasopharynx cancer: 0.092% (0.604%)	Cataract: 0.255% (0.608%)
Encephalitis: 0.153% (0.57%)	Lymphatic filariasis: 0.053% (0.334%)	Nonalcoholic fatty liver disease including cirrhosis: 0.09% (0.591%)	Other malignant neoplasms: 0.239% (0.572%)
Neural tube defects: 0.144% (0.535%)	Cocaine use disorders: 0.052% (0.325%)	Liver cancer due to alcohol use: 0.087% (0.57%)	Cirrhosis due to other causes: 0.235% (0.562%)
Adverse effects of medical treatment: 0.141% (0.524%)	Indirect maternal deaths: 0.05% (0.315%)	Benign prostatic hyperplasia: 0.085% (0.554%)	Osteoarthritis hand: 0.232% (0.555%)
Other chronic respiratory diseases: 0.13% (0.486%)	Maternal abortion and miscarriage: 0.045% (0.282%)	Alcoholic cardiomyopathy: 0.077% (0.504%)	Urinary tract infections and interstitial nephritis: 0.221% (0.527%)

Congenital musculoskeletal and limb anomalies: 0.127% (0.473%)	Unintentional firearm injuries: 0.044% (0.272%)	Poisoning by carbon monoxide: 0.048% (0.314%)	Peptic ulcer disease: 0.214% (0.511%)
Scabies: 0.114% (0.426%)	Acne vulgaris: 0.042% (0.262%)	Motor neuron disease: 0.044% (0.285%)	Other neurological disorders: 0.21% (0.502%)
Diabetes mellitus type 1: 0.112% (0.418%)	Maternal obstructed labor and uterine rupture: 0.04% (0.252%)	Hodgkin lymphoma: 0.042% (0.277%)	Bladder cancer: 0.165% (0.395%)
Dengue: 0.108% (0.401%)	Police conflict and executions: 0.037% (0.233%)	Multiple sclerosis: 0.038% (0.25%)	Other vision loss: 0.161% (0.384%)
Electrocution: 0.094% (0.35%)	HIV/AIDS multidrug-resistant tuberculosis without extensive drug resistance: 0.034% (0.213%)	Liver cancer due to NASH (Nonalcoholic steatohepatitis): 0.038% (0.247%)	Other hemoglobinopathies and hemolytic anemias: 0.16% (0.382%)
Acute hepatitis B: 0.091% (0.339%)	Other drug use disorders: 0.034% (0.213%)	Food-borne trematodiases: 0.037% (0.242%)	Interstitial lung disease and pulmonary sarcoidosis: 0.159% (0.381%)
Otitis media: 0.09% (0.333%)	Onchocerciasis: 0.031% (0.195%)	Cutaneous and mucocutaneous leishmaniasis: 0.027% (0.176%)	Acute myeloid leukemia: 0.151% (0.361%)
Down syndrome: 0.087% (0.324%)	Anorexia nervosa: 0.03% (0.189%)	Liver cancer due to other causes: 0.025% (0.167%)	Pancreatitis: 0.15% (0.359%)
Other chromosomal abnormalities: 0.085% (0.315%)	Ectopic pregnancy: 0.028% (0.174%)	Seborrhoeic dermatitis: 0.013% (0.084%)	Kidney cancer: 0.145% (0.347%)
Sickle cell trait: 0.085% (0.315%)	Polycystic ovarian syndrome: 0.023% (0.146%)	Foreign body in eyes: 0.013% (0.082%)	Gallbladder and biliary tract cancer: 0.143% (0.342%)
Poisoning by other means: 0.084% (0.311%)	Cannabis use disorders: 0.022% (0.14%)	Extensively drug-resistant tuberculosis: 0.009% (0.057%)	Other digestive diseases: 0.126% (0.302%)
Acute hepatitis A: 0.083% (0.311%)	Female infertility: 0.022% (0.14%)	Genital herpes: 0.008% (0.051%)	Aortic aneurysm: 0.122% (0.292%)
Sudden infant death syndrome: 0.082% (0.306%)	Testicular cancer: 0.021% (0.131%)	Pruritus: 0.004% (0.027%)	Liver cancer due to hepatitis C: 0.121% (0.29%)
Thalassemias trait: 0.076% (0.282%)	Late maternal deaths: 0.017% (0.108%)	Cystic echinococcosis: 0.003% (0.023%)	Rheumatoid arthritis: 0.119% (0.285%)
Fungal skin diseases: 0.071% (0.265%)	Other sexually transmitted infections: 0.017% (0.104%)	Leprosy: 0.001% (0.004%)	Environmental heat and cold exposure: 0.113% (0.271%)
Urogenital congenital anomalies: 0.056% (0.207%)	Male infertility: 0.012% (0.073%)		Other sense organ diseases: 0.107% (0.257%)
Tetanus: 0.044% (0.165%)	Trichuriasis: 0.01% (0.063%)		Gastritis and duodenitis: 0.105% (0.25%)
Other road injuries: 0.043% (0.159%)	Trichomoniasis: 0.009% (0.053%)		Multiple myeloma: 0.101% (0.24%)
Vitamin A deficiency: 0.039% (0.144%)	Chlamydial infection: 0.007% (0.041%)		Inguinal, femoral, and abdominal hernia: 0.086% (0.205%)
Thalassemias: 0.036% (0.133%)	Alopecia areata: 0.004% (0.026%)		Nonrheumatic calcific aortic valve disease: 0.084% (0.201%)
Nonvenomous animal contact: 0.032% (0.118%)	Maternal deaths aggravated by HIV/AIDS: 0.003% (0.021%)		Endocarditis: 0.084% (0.2%)
Rabies: 0.031% (0.116%)	African trypanosomiasis: 0.003% (0.019%)		Soft tissue and other extraosseous sarcomas: 0.08% (0.19%)
Paratyphoid fever: 0.029% (0.107%)	Gonococcal infection: 0.003% (0.017%)		Other benign and in situ neoplasms: 0.076% (0.182%)
Ascariasis: 0.029% (0.107%)	HIV/AIDS extensively drug-resistant tuberculosis: 0.001% (0.009%)		Other urinary diseases: 0.075% (0.18%)
Orofacial clefts: %0.02% (0.074)	Klinefelter syndrome: 0% (0.001%)		Osteoarthritis other: 0.074% (0.178%)

G6PD (Glucose-6-phosphate dehydrogenase) deficiency: 0.02% (0.074%)	Ebola: 0% (0%)		Lower extremity peripheral arterial disease: 0.067% (0.159%)
Hookworm disease: 0.016% (0.061%)	Guinea worm disease: 0% (0%)		Malignant skin melanoma: 0.065% (0.156%)
Burkitt lymphoma: 0.013% (0.05%)	Benign and in situ cervical and uterine neoplasms: 0% (0%)		Other leukemia: 0.065% (0.155%)
Acute hepatitis E: 0.013% (0.047%)	Latent tuberculosis infection: 0% (0%)		Pyoderma: 0.064% (0.153%)
Neuroblastoma and other peripheral nervous cell tumors: 0.012% (0.044%)	Benign and in situ intestinal neoplasms: 0% (0%)		Gout: 0.064% (0.153%)
Visceral leishmaniasis: 0.012% (0.044%)			Vascular intestinal disorders: 0.062% (0.147%)
Diphtheria: 0.012% (0.044%)			Other nutritional deficiencies: 0.06% (0.144%)
Acute hepatitis C: 0.012% (0.043%)			Thyroid cancer: 0.056% (0.133%)
Yellow fever: 0.011% (0.041%)			Appendicitis: 0.052% (0.124%)
Hepatoblastoma: 0.011% (0.041%)			Other skin and subcutaneous diseases: 0.049% (0.117%)
Retinoblastoma: 0.01% (0.038%)			Nonmelanoma skin cancer (squamous-cell carcinoma): 0.048% (0.114%)
Caries of deciduous teeth: 0.007% (0.026%)			Myelodysplastic, myeloproliferative, and other hematopoietic neoplasms: 0.044% (0.104%)
Other intestinal infectious diseases: 0.003% (0.013%)			Ulcerative colitis: 0.043% (0.103%)
G6PD (Glucose-6-phosphate dehydrogenase) trait: 0.001% (0.002%)			Osteoarthritis hip: 0.043% (0.102%)
Turner syndrome: 0% (0.001%)			Cysticercosis: 0.042% (0.101%)
Zika virus: 0% (0%)			Cellulitis: 0.04% (0.097%)
			Decubitus ulcer: 0.039% (0.094%)
			Nonrheumatic degenerative mitral valve disease: 0.037% (0.087%)
			Urolithiasis: 0.035% (0.085%)
			Chronic lymphoid leukemia: 0.035% (0.084%)
			Varicella and herpes zoster - 0.03 (0.071)
			Chronic myeloid leukemia: 0.028% (0.068%)
			Foreign body in other body part: 0.026% (0.061%)
			Pulmonary arterial hypertension: 0.025% (0.06%)
			Glaucoma: 0.024% (0.058%)
			Myocarditis: 0.023% (0.055%)
			Mesothelioma: 0.022% (0.053%)
			Age-related macular degeneration: 0.018% (0.044%)
			Genital prolapse: 0.015% (0.037%)

			Crohn's disease: 0.015% (0.035%)
			Silicosis: 0.012% (0.03%)
			Acute glomerulonephritis: 0.012% (0.028%)
			Chagas disease: 0.01% (0.024%)
			Uterine fibroids: 0.008% (0.019%)
			Other eye cancers: 0.007% (0.018%)
			Trachoma: 0.005% (0.012%)
			Other pneumoconiosis: 0.002% (0.006%)
			Coal workers pneumoconiosis: 0.002% (0.005%)
			Asbestosis: 0.002% (0.005%)
			Other nonrheumatic valve diseases: 0.002% (0.004%)
			Nonmelanoma skin cancer (basal-cell carcinoma): 0% (0%)

We base our cluster analysis on the 2023 GBD.[2] A potential problem for our analysis is if a high degree of volatility characterizes how diseases are allocated to clusters. To investigate this, we applied our clustering algorithm to all years from 1990 onward. Prior to 2001 the clustering algorithm still identifies four clusters, but it has two that capture infant diseases, and it does not separately identify a late adult category, tending instead to merge the late adult and ageing-related cluster together (with the overall cluster showing an increasing trend with age). From 2001 our four clusters—infant, adult (early), adult (late), and ageing-related—have emerged and remain broadly stable over the next 20 years (see Figure A1). As well as the lifetime pattern being highly stable, the composite diseases also remain broadly constant. The 2001 centroid for ageing-related diseases contains 90% of the diseases that are in the 2023 cluster and always remains greater than 90% in every subsequent year.

Supplementary Figure 1: Robustness of Disease Clusters 2001–2023

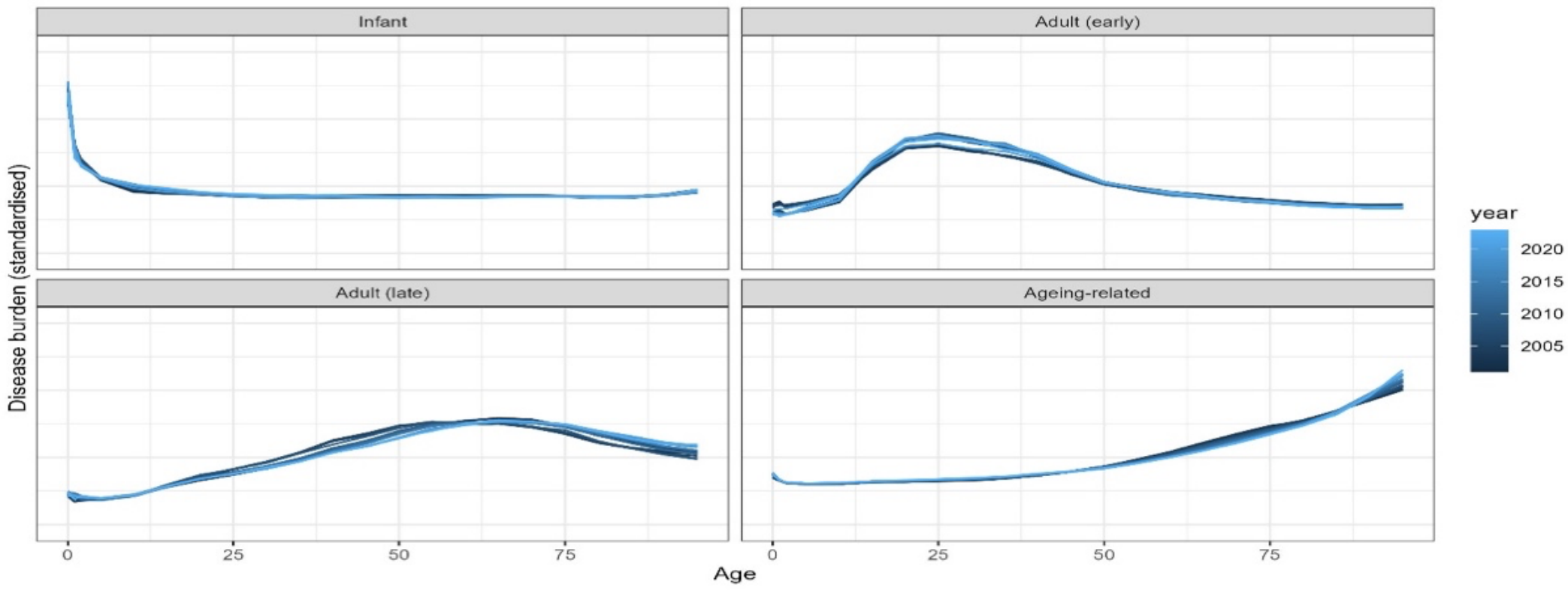

Figure shows estimated disease burdens by age for four disease clusters estimated for each year of GBD data availability to assess robustness across years.

Results without COVID-19

Our initial analysis of this topic was performed using GBD 2021. This year was still significantly influenced by COVID, and because the mortality rate connected with COVID increased significantly with age our clustering algorithm allocated COVID to the ageing-related cluster. Because we did not want our findings to be influenced by the pandemic, we overrode the algorithm and performed our analysis keeping COVID as a separate category. Even excluding COVID we found that ageing-related diseases

represented the main current disease burden and the greatest lifetime health burden for a newborn anywhere in the world and experienced substantial increasing returns.

Updating our results with 2023 data, our algorithm still classifies COVID as an ageing-related disease but the incidence of COVID is now much reduced (see Figure B1 and Table B1). Therefore, for simplicity we no longer treat COVID as a separate category and follow the clustering algorithm's recommendation to include it in the ageing-related category. Our results remain the same whether we do or do not include it in the ageing-related cluster.

Supplementary Figure 2: Mortality and Disability Burden by Cluster, 2023

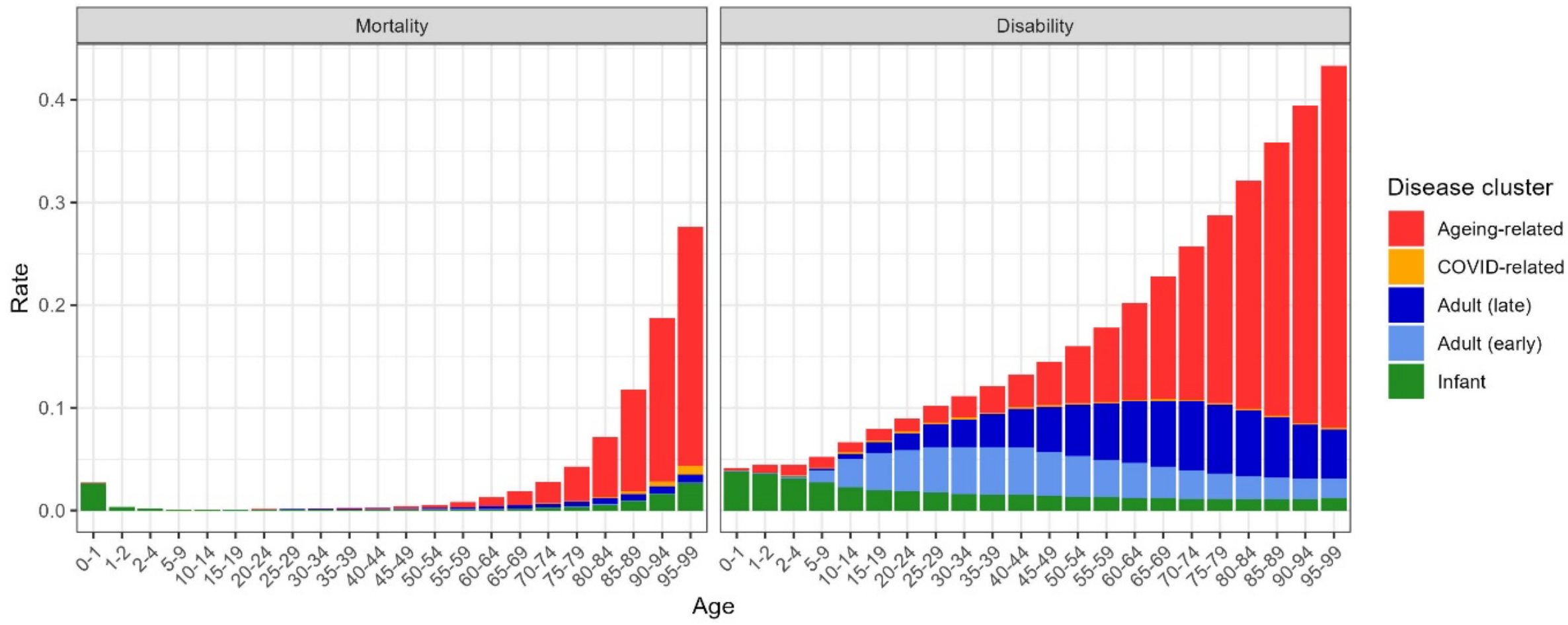

Supplementary Table 4: Current Global Disease Burden by Cluster, 2023 (percent)

Disease Cluster	Global DALYs	Global YLDs	Global YLLs	High Income DALYs	High Income YLDs	High Income YLLs	Upper Middle Income DALYs	Upper Middle Income YLDs	Upper Middle Income YLLs	Lower Middle Income DALYs	Lower Middle Income YLDs	Lower Middle Income YLLs	Low Income DALYs	Low Income YLDs	Low Income YLLs
Ageing-related	0.41	0.34	0.45	0.54	0.60	0.65	0.50	0.58	0.58	0.34	0.41	0.38	0.19	0.31	0.18
COVID-related	0.01	0.01	0.01	0.01	0.03	0.01	0.02	0.02	0.02	0.01	0.03	0.01	0.01	0.05	0.01
Adult (late)	0.15	0.23	0.11	0.20	0.15	0.16	0.19	0.15	0.15	0.12	0.15	0.08	0.09	0.08	0.07
Adult (early)	0.16	0.27	0.10	0.17	0.14	0.11	0.16	0.12	0.09	0.15	0.12	0.09	0.17	0.22	0.12
Infant	0.27	0.15	0.33	0.07	0.08	0.08	0.13	0.13	0.15	0.37	0.29	0.44	0.55	0.33	0.62

Measure

Proportion of current disease burden: 0.2, 0.4, 0.6
